# Bacteria-related changes in host DNA methylation and the risk for CRC

**DOI:** 10.1080/19490976.2020.1800898

**Published:** 2020-09-15

**Authors:** Iradj Sobhani, Hugo Rotkopf, Khashayarsha Khazaie

**Affiliations:** a Head of the Department of Gastroenterology, Consultant in GI Oncology, Hopital Henri Mondor, APHP. Créteil-France; Head of the Research Team EC2M3, Université Paris-Est Créteil (UPEC), Créteil, France; b Department of Gastroenterology Hospital Henri Mondor, APHP. Créteil-France; Member of Research Team EC2M3, Université Paris-Est Créteil (UPEC). Créteil, France; c Department of Immunology, SW Rochester, MN

**Keywords:** Colon, cancer, microbiota, mutation, methylation

## Abstract

Colorectal cancer (CRC) is the second most common cause of cancer deaths in men and women combined. Colon-tumor growth is multistage and the result of the accumulation of spontaneous mutations and epigenetic events that silence tumor-suppressor genes and activate oncogenes. Environmental factors are primary contributors to these somatic gene alterations, which account for the increase in incidence of CRC in western countries. In recent decades, gut microbiota and their metabolites have been recognized as essential contributing factors to CRC, and now serve as biomarkers for the diagnosis and prognosis of CRC. In the present review, we highlight holistic approaches to understanding how gut microbiota contributes to CRC. We particularly focus herein on bacteria-related changes in host DNA methylation and the risk for CRC.

## Introduction

Colorectal carcinoma (CRC) is one of the three most common cancers, with more than 1.2 million new cases and about 600,000 deaths worldwide every year.^1^ CRC tumors result from an accumulation of DNA alterations that alter key signaling pathways. This forms part of a multistage carcinogenesis process that involves epithelial hyperproliferation, development of aberrant crypt foci (ACF), adenoma to carcinoma transition, tumor invasion, and metastasis. Most CRCs are of a sporadic nature and less than 5% are hereditary,^2^ which suggests that environmental factors are the common underlying cause of CRC carcinogenesis, rather than inherited genetic defects. Environmental exposure is considered an underlying feature of the increasing prevalence of sporadic CRCs in the western world. Although they have been investigated for a long time, exhaustive analysis of environmental factors is a daunting task and appears almost impossible. A major breakthrough has come with the association of gut microbiota (GM) to CRC. The causative role of GM in disease was first illustrated with mouse models, in studies of obesity and nutrition.^3,4^ A community of stool bacteria from obese mice that had been submitted to hyperphagia transmitted obesity to germ-free recipients while they were submitted to normophagia.^4^ GM has now become a focus of investigations as biomarkers for both host and bacteria adaptation to the environment. Ongoing research has linked GM with various diseases, including diabetes, inflammatory bowel disease (IBD), and CRC. This review analyzes GM as a novel tool for studying colon carcinogenesis, focusing on changes in host DNA methylation as a specific etiologic mechanism.

## Mutations in CRC

The genetic basis of cancer was firmly established by the discovery and cloning of the adenomatous polyposis coli (APC) gene, whose loss of function is likely the most common cancer initiator in CRC.^5–9^ This was followed by the recognition that CRC carcinogenesis involves several genetic events and is a multistage process.^10,11^ Individuals that are born with a heterozygous APC deficiency develop familial polyposis (FAP), which, if left untreated, develops into colon cancer. A second form of hereditary colon cancer is caused by defects in the DNA mismatch repair system (MMR). Heritable MMR gene mutations are the primary cause of hereditary nonpolyposis colorectal cancer (HNPCC), which is known as Lynch syndrome.^12^ However, both APC and MMR gene silencing similarly contribute to sporadic CRC development.

**Figure 1.**
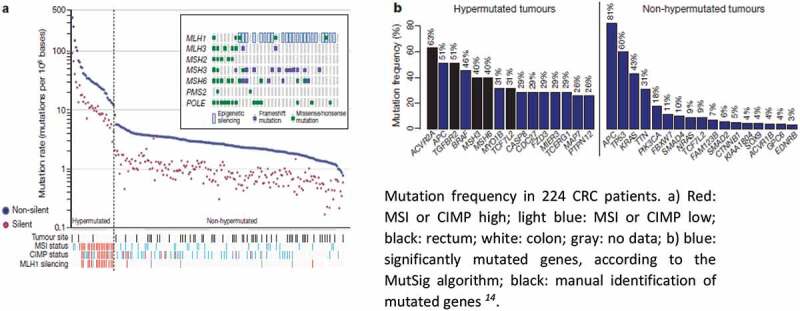
Frequency and type of mutations in human CRC tissues.

Several critical genes and pathways that are essential in CRC initiation and progression have now been described. Accordingly, consensus molecular CRC subtypes have also been described,^13^ and a cancer genome atlas on CRC has been established.^14^ Overall, 24 host genes frequently mutate in CRC, including APC, TP53, SMAD4, PIK3CA, KRAS, ARID1A, SOX9, FAM123B, TGF-β, and DCC. Additionally, MMR genes exhibit recurrent copy-number alterations with drug-targetable amplifications of ERBB2, amplification of IGF2, and recurrent chromosomal translocations with the fusion of NAV2 and WNT-pathway member TCF7L1. Integrative analyses suggest new markers for aggressive CRC and an essential role for MYC-directed transcriptional activation and repression. Particularly, several of these DNA mutations can be detected in precancerous lesions (Table 1). Based on mutation rates within tumor tissues, the Cancer Genome Atlas project^14^ divided CRCs into two groups: those with a mutation rate of <8.24 per 10^6^ (84% of cases, which are designated hypomutated) and remaining cases with mutation rates of >12 per 10^6^ (which are designated hypermutated) (Figure 1). Overall, 16% of CRCs are hypermutated, and most of these fall into the category of tumors with microsatellite instability (MSI), which is either due to mutations in the MMR or promoter methylation of the MLH1 gene (CIMP).^15,16^ By contrast, the majority of CRC tumors have markedly fewer mutations but exhibit chromosomal instability (CIN), which is primarily caused by hereditary or spontaneous inactivating mutations in the APC gene.

**Table 1.** Colorectal carcinogenic pathways and main genetic alterations.

	MSI	Methylation	KRAS	BRAF	TP53
Adenoma-carcinoma sequence	-	±	++	-	+
Serrated pathway	+	++	+	++	±
*De novo* pathway	-	-	+	-	-

### DNA Methylation as a marker of global health and mortality

DNA methylation in palindromic CpG dinucleotides (CpG islands) plays a pivotal role in mammalian genomic stability and gene expression.^17^ In normal physiology, *de novo* methylation events are secondary to gene repression. They serve as a long-term memory of earlier gene expression decisions that have defined cell fate and function, which are intended to prevent gene reactivation in subsequent cell generations.^18,19^ By contrast, the active demethylation of gene regulatory sequences is an integral component of the mechanisms that drive cellular differentiation through gene expression.^20,21^

The covalent bonding of methyl groups at the five positions of cytosine (5mC) is catalyzed by DNA methyltransferases (DNMTs). The addition of methyl groups to unmethylated cytosines, *de novo* methylation, is catalyzed by DNMT3 family enzymes. While *de novo* methylation of CpG islands in gene promoters results in gene silencing, intergenic methylation controls spurious transcription initiation and regulates alternate promoter usage and RNA alternative splicing, which often enhance gene expression. After establishing DNA methylation, the methylation pattern is maintained during DNA replication by the maintenance DNA methyltransferase DNMT1, through a process that is guided by hemi-methylated DNA and histone marks. DNMT1 plays a critical role in preserving epigenetic memory during cell division. The removal of methylation is important for cellular differentiation and is mediated by ten-eleven translocation (TET) proteins.^20,21^ Active genome-wide DNA demethylation allows for the pluripotency of germ cells during the early stages of embryonic development, which is followed by remethylation prior to birth in males and after birth in females.^17^ Likewise, TET proteins and global demethylation have been linked to the dedifferentiation process in cancer.^22^ Passive demethylation occurs when the DNMT1 function is inhibited during DNA replication, or when there is a shortage of cofactors like S-adenosylmethyonine (SAM).

Based on thorough comparative analysis of different CpG island sets from different tissues and age spectra, DNA methylation has emerged as a promising estimate of age and age-related conditions.^23,24^ The aging process has been associated with what appears to be stochastic changes in patterns of DNA methylation in multiple tissues, which begin in the embryo and slowly continue over time. This process includes *de novo* methylation of the CpG island set that is bound by the polycomb repressor and demethylation of other large areas of the genome, which is reviewed by Dor and Cedar.^24^ By comparing methylated loci under physiological and pathological conditions from 30 different tissues and cell types from adults and children, Horvath^25^ demonstrated that DNA methylation events occur in a highly specific manner, and form tissue-specific patterns of biological aging. Similarly, Hannum^26^ derived a highly accurate age estimator on the basis of 71 CpGs from adult blood DNA, which became a predictor of human health commonly known as “Hannum’s clock”. Subsequently, Horvath used a weighted average of 10 clinical blood biomarkers of chronological age to select blood CpGs that estimate biological age and predict mortality.^27^

As an output, methylation markers can be used to estimate disease-related age and mortality. Indeed, all-cause mortality is widely studied in association with DNA methylation age. In a study of 5,124 older individuals from the Northeast US, the Framingham Heart Study demonstrated that methylation-related age was associated with mortality,^28^ which was corroborated by newer studies.^29,30^ Additionally, a study of 86 Danish twins found that the twin with the higher DNA methylation age at baseline displayed a 69% probability of dying first, over a 10-year follow-up period. In a German population-based cohort, 5-year higher DNA methylation age was associated with a 23% (CI = 1.10–1.38) and 10% (CI = 0.94–1.29) increased risk of all-cause mortality, respectively, after adjustment for technical and clinical confounders.^31.^

Thus, applying these age estimators to the general population can predict outliers; that is to say, it can identify individuals that are associated with a greater number of age-related conditions and diseases, including cancer. Consequently, DNA methylation-based measures of biological aging have been associated with increased cancer risk and shorter cancer survival, independently of major health risk factors.^32,33^ Since DNA methylation levels that are associated with cellular senescence relate to cancer occurrence, the CancerClock predictive model of age was recently proposed, which includes methylations of 282 sites from different tumor samples.^34^

### DNA Methylation in CRC

Changes in DNA methylation patterns and histone modifications play key pathophysiological roles in the etiology of cancer, as discussed below. Hypermethylation silences the expression of tumor-suppressor genes and directly impact cancer initiation and progression. Much of the research on the impact of hypermethylation in cancer has focused on CRC. In 1999, Toyota et al.^35^ reported that some CRCs display a significantly high frequency of aberrant DNA methylation in specific CpG islands, named the CpG island methylator phenotype (CIMP). Serrated polyps, which are the immediate precursors to CIMP tumors, evolve through activation of the MAPK-ERK pathway by BRAF/KRAS mutations and are distinguished by methylation-mediated transcriptional inactivation of various genes that belong to the β-catenin/WNT pathway (SFRP family, CDX2, and MCC), the insulin-like growth factor signaling pathway (IGFBP7), cell-cycle control proteins (CDKN2A), and the DNA mismatch repair (MLH-1) family^36,37^, CIMP-positive CRC shows DNA hypermethylation of the MLH1 mismatch repair gene, ^38^ and is highly enriched for the BRAF V600E-activating mutation. BRAF is an upstream activator of the DNMT genes.^39^ More recent studies indicate that DNMT1, DNMT3B, and EZH2 histone (H3K27) methyltransferase are all upregulated in both hereditary and sporadic MSI tumors.^40^

Promoter hypermethylation of specific tumor-suppressor genes is emerging as a new biomarker of CRC,^41,42^ including CDKN2A (p16INK4A and p14ARF)^43,44^ and APC,^45^ as reviewed by Okugawa and colleagues.^3,6,46^ A higher incidence of hypermethylation and a low expression of the secreted frizzled-related proteins (SFRP) genes, which are negative regulators of the WNT pathway, is observed in the normal colonic mucosa of patients with CRC, in comparison with healthy individuals.^47^ Higher methylation levels of age-related markers, such as *ESR1* and *MyD88*, were observed in the normal colonic mucosa of patients with CIMP-positive CRC, compared to individuals without CRC. Genome-wide DNA methylation analysis has revealed that the gene methylation levels involved in the metabolic pathways of carbohydrates, lipids, and amino acids are significantly different among normal colonic mucosa specimens that are obtained from patients with CRC and controls. Extensive sequencing data obtained from primary and metastatic tumor tissues reveal that genetic mutations alone are insufficient for predicting metastatic potential, while epigenetic changes contribute to the acquisition of additional properties that are necessary for cancer metastasis.^48,49^ Thus, DNA methylation is emerging as a promising area of investigation for cancer prediction, prevention, and therapy.

Various environmental factors have been investigated for their association with specific patterns of DNA methylation and cancer predisposition or incidence. Among these, chronic inflammation is recognized as a significant inducer of aberrant DNA methylation, as demonstrated by the analysis of non-cancerous tissues, such as colonic mucosae in ulcerative colitis.^50^ Observations in mouse models are starting to provide mechanistic cause and effect insights into how inflammation and DNA methylation contribute to the etiology of CRC. In a mouse colitis model induced by dextran sodium sulfate, aberrant CpG island methylation in colonic epithelial cells was shown to accumulate gradually,^51^ and to be heritable.^52^ Dnmt3b, which is frequently activated in human tumors, promotes tumor development in APCMin/+ mice,^53,54^ establishing a cause and effect relation. Interestingly, the transgenic expression of Dnmt3b, in the mouse colon initiates *de novo* DNA methylation of genes that resemble those becoming methylated in human colon cancer. This indicates that aberrant methylation in cancer targets specific sequences and is not random.^55^ In our recent studies, described in more detail below, transfer of gut microbiota from CRC patients to germ free mice produced similar changes in methylation patterns of oncogenes and tumor suppressor genes in the mouse colon.^56^ Drugs for the modulation of DNA methylation have also shown preclinical promise in terms of slowing tumor progression.^57^ Thus, mouse models are a valuable tool for understanding the link between the environment, and in particular the microbiome, and host methylation events that increase CRC risk and treatment outcomes.

### Significance of hypomethylation

DNA hypomethylation refers to the loss of the methyl group in the 5-methycytosine nucleotide. The first evidence that demethylation results in the reactivation of silenced genes *in vivo* came from the treatment of cultured mammalian cells with the demethylating agent deoxyazacytidine, as well as from mutagenesis-driven selection of temperature-sensitive mutants in DNA hypomethylation.^58^ There is accumulating evidence that DNA hypomethylation and gene reactivation play a major role in the etiology of aging and cancer. While CpGs, at which methylation significantly associates with transcription (eCpG dinucleotides) show heterogeneous distribution, they demonstrate aberrant hypomethylation in aging^59^ and cancers.^60^ In normal physiology, aging is known to reduce global methylation of DNA. Indeed, centenarian DNA has a lower DNA methylation content throughout the genome, with more hypomethylated CpGs in promoter, exonic, intronic, and intergenic regions, compared to newborn DNA.^61^ Interestingly, this global hypomethylation was associated with a greater level of DNA methylation in the CpG island promoters of a few genes^61^ that have similar features to the cancer epigenome. Some examples of hypomethylation and gene reactivation in cancer include the loss of imprinting in IGF2 as an early event in CRC carcinogenesis.^62^ Another example is MAGE gene families, which are physiologically repressed by promoter methylation in normal somatic tissues and activated through promoter hypomethylation in several types of cancers.^63^ Several other examples of the hypomethylation of key procarcinogenic genes and their overexpression have been reported,^64^ such as the BCL2 gene in lymphocytic lymphoma, *RRAS* in gastric cancers, *MAGE* family genes, and GPR17 in lung, head, and neck cancers.^57^ The association of DNA hypomethylation with high HIF-1α expression levels has critical implications for energy metabolism, angiogenesis, cell survival, and tumor invasion.^57^ The HIF-1α promoter is an auto-transactivating gene that enables the HIF-1α protein to bind to its own promoter, which explains why hypomethylation of this gene activates tumor growth. Thus, widespread DNA hypomethylation and focal hypermethylation characterize both aging and cancer epigenomes.

**Figure 2.**
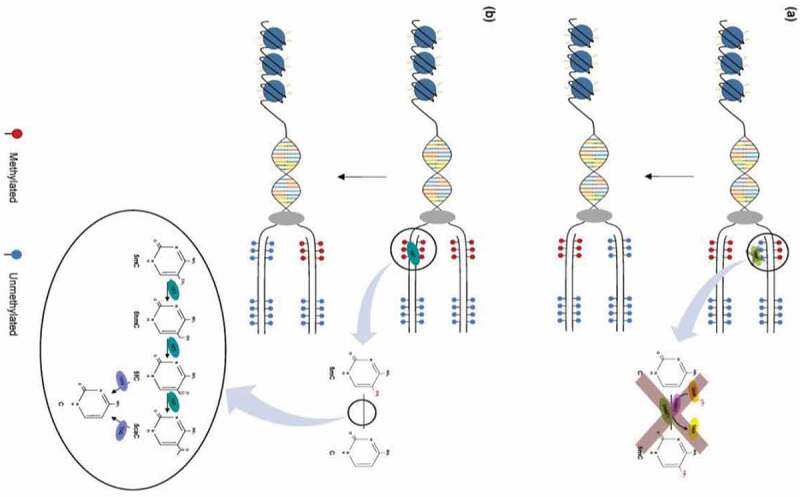
Mechanisms of DNA demethylation. (a) Passive demethylation. This process occurs during replication, wherein one or more limiting factors (i.e., compromised DNMT function, absence of SAM) prevents methylation maintenance and results in the subsequent loss of 5mC residues. (b) Active demethylation. The figure shows TET enzymes (TET1, TET2, or TET3) (teal) catalyzing stepwise oxidation of 5mC, which is first converted into 5-hydoxymethylcytosine (5hmC), further oxidized into 5-formylcytosine (5fC), and finally converted into 5-carbocylcytosine (5caC). 5fC and 5caC intermediates can be recognized and removed by thymine DNA glycosylase (TDG) (violet). They are then replaced with an unmethylated cytosine nucleotide to complete the base excision repair (BER) process (figure from ref).^57^

DNA hypomethylation can produce chromosomal instability, loss of imprinting, and the reactivation of endogenous transposable elements and retroviruses.^65^ However, the experimental outcomes of hypomethylation can vary, depending on the approach used. While treatment with 5-aza-deoxycytidine reduces tumor burden in mouse models of hereditary polyposis,^66^ DNMT1 deficiency predisposes mice to developing lymphomas.^67^ Additional studies indicate that, while DNA hypermethylation of specific loci drives CRC tumorigenesis,^68,69^ genome-wide hypomethylation is associated with chromosomal instability in CRC.^70^ Thus, mouse models are also beginning to demonstrate the nonrandomness of DNA hypomethylation and its likely association with aging and cancer predisposition (Figure 2; Table 2).

**Table 2.** Hallmarks of cancer and examples of genes silenced by aberrant methylation.

Hallmark	Gene	Gene Function	
WNT pathway	WIF1, SFRP	Inhibit the WNT pathway	
Self-sufficiency in growth signals	RASSF1A	Regulation of Ras pathway	
Mismatch repair	hMLH1	Failure to correct mutations	
Evading apoptosis	Caspase-8	Initiation of apoptosis	
Insensitivity to antigrowth signals	p16/CDKN2A	Cyclin-kinase inhibitor	
Tissue invasion and metastasis	VHL (Von Hippel-Lindau)	Suppression of metastasis	
Sustained angiogenesis	VEGF-2	Crucial for angiogenesis	
Limitless replicative potential	RB (Retinoblastoma)	Cell-cycle regulation Deregulated metabolism	
Immune evasion	Protection via T helper DNMT blockers improve survival Beta Catenin		

The disruption of epigenetic mechanisms allows tumor cells to gain hallmark properties in the same manner as genetic mutations. Promoter hypermethylation leads to loss of gene function.

**Figure 3.**
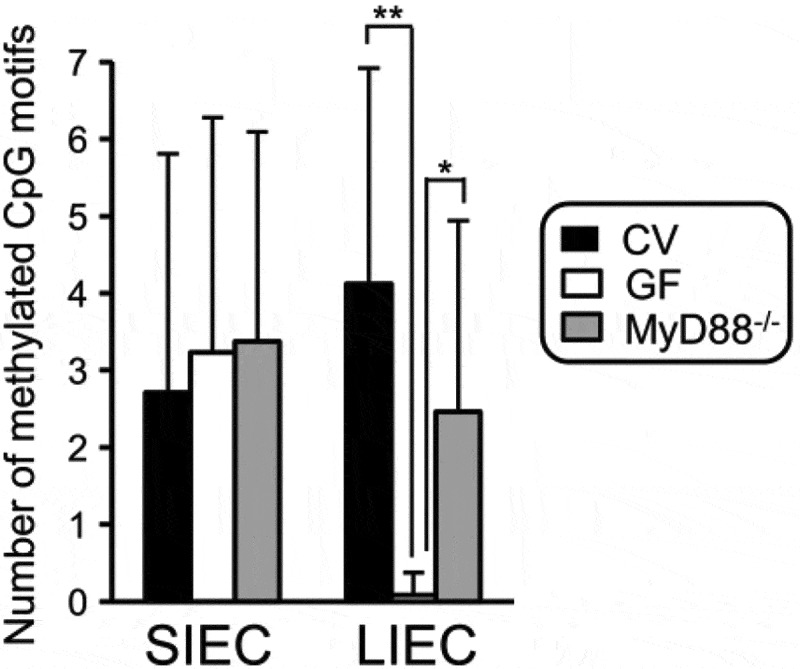
IEC: intestinal epithelial cell; SIEC: small; IECL: IEC large intestine; CV: conventional; GF: germ-free; IEC: intestinal epithelial cell; SIEC:small; LIEC: large intestine; CV:conventional; GF: germ-free; from Takashi et al Ref. ^94^.

### Microbes and CRC

The characterization of colonic gut microbiota that is causatively linked to CRC raises two main difficulties. The immense number of microbes (10^12 to14^/gr) and proportion of anaerobes in the stool make it difficult to culture them all. Thus, GM is usually characterized by using 16S rRNA and whole metagenomic sequencing procedures. We previously showed, by using principal component analyses, that GM from CRC patients and controls^71^ display statistically significant separation, as also reported by others.^72^ However, a variety of bacterial species, not a single microbe, including commensal and virulent bacteria, were associated with tumors. Also, based on the characterization of tissue-adherent bacteria, we found that microbial organization and networking were different in normal and tumor tissues, even in the same individual.^71,73^ Some microbial species were more frequently present in tumor tissue than in homologous normal tissue throughout the entire progression of a disease, from early tumor development to metastasis.^74^ At this time, however, cause and effect have not been established.

To understand the mechanisms through which microbiota may be involved in colon carcinogenesis, it was first necessary to clarify the “chicken or egg” relationship. Several groups have shown that unselected bacteria from CRC patients’ microbiota can initiate and/or promote colon carcinogenesis.^56,75^ As effective causative evidence, PEG, a laxative component that is known to impede the adherence of bacteria to the mucosa, was shown to reduce the development of aberrant Crypt foci (ACF) in experimental mouse models.^56^ This was also shown in humans, as demonstrated in a randomized double-blind placebo trial.^76^ In all these studies, the carcinogenic effect of the microbiota was associated with various abnormalities, such as epithelial cell hyperproliferation, inflammatory cytokines release, and inflammatory and immune cell infiltration within the colonic mucosa. In addition, injuries in the mucosa were found to be significantly linked to microbial composition, regardless of whether they were associated with precancerous and cancerous lesions. Such abnormalities within tissues have been observed in the normal macroscopic colonic mucosa of various other emerging diseases, such as obesity, IBD, and insulin resistance.^73,77,78^ Thus, it became necessary to address how microbial-related effects could be specific to CRC.

Results from studies done based on single bacterium–cell or bacterium–tissue interactions have enriched our knowledge of how microbiota impact host homeostasis or promote pathology. Disruption of cell–cell junctions in the colonic mucosa and enhanced mutations in the host DNA have been attributed to *Escherichia coli* strains, *Bacteroides fragilis*, and *Fusobacterium nucleatum.*^78^^,79^However, the putative action of each bacterium remains questionable, due to the large number ofbacteria and difficulties in distinguishing direct and indirect effects, such as DNA damage,chromosomal instability, inflammation, and metabolism.^80^ One attractive approach to moving from individual species to more complex microbiota is to focus on microbial metabolites, such as H2S^81,82^ and secondary bile acids^83^, both of which can cause DNA damage as procarcinogenic agents and alter immunity.^78^

### Virulent microbes and host DNA mutations

Multiple studies analyzing colonic mucosa samples from CRC patients have reported an association between virulent pro-inflammatory bacteria, such as the pathogenic strains of *E. coli*, and CRC through the depletion of MMR protein network in host cells. For example, enteropathogenic *E. Coli* (EPEC) produces the EspF toxin that depletes MMR proteins MSH-2 and MLH-1 in cultured colonic cells^84^ Bacteria–cell interaction leads to the rapid accumulation of spontaneous somatic mutations throughout the genome, particularly in long repeated sequences of 1–4 nucleotides, which are termed microsatellites.^15,85^ In addition to causing microsatellite instability (MSI), MMR disruption enhances the somatic mutation of tumor-suppressor genes, such as APC and P53, which are mutated in the majority of CRCs. Whether EPEC induces somatic mutations in MMR genes themselves has not yet been established.

*E. coli* is divided into various phylotypes (A, B1, B2, D, E, and F). Although significant levels of *E. coli* were detected in CRC tissues, compared to healthy colonic tissues, the *E. coli* from CRC cases (57%) belonged to different phylotypes.^86^ All Phylotype A *E. coli* strains are commensal, but B2 is the most prevalent haplotype in feces. Based on virulence factors, all the strains belonging to the phylotypes B2, D (with an exception), E, and F cluster together.^87^ Whether this is implicated in colon carcinogenesis remains unclear. However, there is a high likelihood that virulent bacteria work as consortia rather than alone. For example, colibactin produced by pks^+^ *E*. coli works synergistically with fragilysin a toxin produced by enterotoxigenic *Bacteroides fragilis* (ETBF), to damage DNA in colonocytes and stimulate pro-inflammatory response in mice.^88^

Overall, and at the genus level, 20–30 bacterial genera have been found to be significantly different between CRC patients and controls, and at the OTU level, more than 80 genera have marked differences.^56,89^ However, fewer shifts were observed in a Chinese cohort.^90^ We reported noticeable shifts in Prevotella/Bacteroides in the stool samples of CRC patients versus controls.^71^ Lower diversity in CRC patients’ stool was reported in most studies, with enrichment of *Fusobacterium, Porphyromonas, P. micra*, and *B. fragilis*.^78,79,91^ This suggests that, for each of these bacteria, various species may preferentially be linked with CRC, which indicates that it is highly unlikely that a single causative bacterium with one or several mutagenic effects on the colonic mucosa will be identified. It has not yet been clarified whether bacteria overgrowth exerts a direct effect or damages in the colonic mucosa result from chronic inflammation. The growth of virulent bacteria seems to be favored by meat-enriched diets,^92^ however the impact of tumor growth and changes in immune response on enrichment of such harmful strains needs more investigation.

### Commensal habitants and the significance of host DNA methylation

Commensal bacteria inhabit the colon, where they contribute to host nutrition, prevent pathogen colonization, and are involved in the regulation of various physiological functions. DNA methylation is one of the tools by which they influence physiological functions or cause pathology. Exposure to GM drives distinct methylome and transcriptome changes in intestinal epithelial cells during human post-natal development.^93^ In adults, decreased expression of specific toll-like receptors (TLRs) in colonocytes limits their responsiveness to microbiota, which prevents autoimmunity.^79^ Increased expression of TLR4 and an elevated response to commensal organisms are observed in patients with IBD.

Takashi et al.^94^ investigated the methylation level of the TLR4 gene in the intestinal epithelial cells (IEC) of conventional mice (CV) and compared results to those of germ-free (GF) mice that did not have intestinal commensal bacteria. First, the methylation level of the TLR4 gene was significantly lower in the colonocytes of GF mice than in those of CV. Second, DNA methylation in the TLR4 gene promotor in CV mice was higher in IECs than in splenic cells in CV. Third, this methylation was dependent on the differentiation state of the IECs, as the differentiated IEC population display greater methylation and lower expression of the TLR4 gene than the undifferentiated population. Fourth, overexpression of CDX2, which is a transcription factor that is required for the development of the intestine, decreased the methylation level of the TLR4 gene promoter and increased gene expression and responsiveness to TLR4 liganchanges in intestinal epithelial cells during in the small intestine, which does not host commensal bacteria, was similar in both CV and GF mice.^94^ These findings show that although IECs are continuously exposed to commensal bacteria, they remain relatively insensitive to them, which avoids an excessive inflammatory reaction in the colonic mucosa. Human IEC lines are hyperresponsive to LPS *in vitro* because of the down-regulation of TLR4 gene expression, which is silenced through several epigenetic mechanisms. By controlling epigenetic modification of the host genes in the large intestine, the commensal bacteria maintain intestinal symbiosis and may contribute to healthy mild inflammation within the mucosa. Thus, epigenetic-mediated mechanisms, including DNA methylation and histone deacetylation, are suggested as the main pathway by which commensal bacteria regulate colonocyte maturation and responsiveness to the microbiome.^50^

Moreover, altered DNA methylation in gut epithelial cells can have pathological consequences. A well-known example is the hypermethylation of MMR genes that are predisposed to sporadic MSI colon cancer. Hypermethylation of CpG islands in the promotor of hMLH1, which is one of the main functional genes of the MMR system, mimics MMR system failure and resembles Lynch syndrome.^95^ Thus, we suggest that DNA methylation plays a pivotal role in maintaining symbiosis between the mucosa and commensal bacteria in the intestine regulating host DNA mutations and repair that are caused by invasive and virulent bacteria.

### Microbiota-induced methylation and immune response to CRC

Although CRC was the first neoplasia found to be under immune surveillance,^96^ it is largely resistant to immunotherapy.^97^ This can be partly explained by the immune suppressive microenvironment of CRC tumors, which includes expression of immune checkpoint molecules^98,99^ and a local T-cell suppressive inflammatory infiltrate.^100^ Paradoxically, DNA hypermutation that occurs in relatively rare hereditary MMR or sporadic MSI tumors improves antitumor immune responses, compared to more frequent CIN tumors, which have fewer mutations. DNA hypermutation generates neoantigens^101^ and enhances T-cell responses to the tumor, which increases the densities of tumor-infiltrating T-cells^102-104^ that respond to mutation. These findings have led to the description of an Immunoscore, which measures tumor-infiltrating T-cell density as a predictive marker of cancer outcome, and its incorporation into the classical CRC tumor staging.^105,106^ Following this rationale, pharmacologic demethylation of tumor-associated DNA may boost protective T-cell responses through the activation of endogenous retroviruses and cancer-testis antigens, which are normally suppressed in somatic cells. Activation of these genes can give rise to neoantigens in treated cells, which increase the T-cell immunosurveillance capability of the host and thus improve clinical outcomes.^107^

Changes in the DNA methylation patterns in the blood (PBMC and free circulating DNA) are emerging as a characteristic feature of CRC. In a recent study, we linked changes in methylation patterns of blood mononuclear cells with CRC-associated microbiota.^56^ We found that several gene promoters, including SFRP1, 2, and 3, PENK, NPY, ALX4, SEPT9, and WIF1, were hypermethylated in the CRC tumoral tissues and blood, compared to healthy donor tissues and blood. Based on these reults, an easily reproducible blood test was developed, and validated in a large cohort of 1,000 individuals, including 187 with advanced adenomas or invasive cancer in their colon and coloscopy, with no significant abnormalities in the remaining cases. The blood test that included methylation levels of WIF1, PENK, and NPY genes, which is called the cumulative methylation index (CMI), was closely associated with both CRC and dysbiosis. Further, CMI appeared to be an independent risk factor for CRC diagnosis, as shown by multivariate analysis that included a fecal immunochemical blood test using a mass-screening program, too. Overall, individuals with higher CMI in their blood were identified as having a greater abundance of pro-epigenomic bacteria in their stools. To relate methylation changes in the host with gut microbiota, we took fresh fecal samples from nine subjects with normal colonoscopy and from nine sex- and age-matched CRC patients, and transferred these microbiota (FMT) into germ-free (GF) mice. To accelerate any oncogenic events produced by the microbiota, separate groups of these mice were treated with low levels of AOM. Fecal and colonic mucosa features were measured, using histopathological and molecular techniques. Between seven and 14 weeks after FMT, CRC-associated microbiota induced higher numbers of aberrant crypt foci (ACF) as compared to healthy donor microbiota. The increase in ACF incidence corresponded to higher levels of hypermethylated genes in the colonic mucosa of the mice that received CRC-microbiota relative to healthy control microbiota recipients, but no significant difference in DNA mutation was observed between the animal groups. Thus, we conclude that the panel of bacteria that is associated with high levels of CMI can be potentially used for screening and therapy surveillance in CRC patients.^56^

Other independent studies have found correlations between DNA methylation patterns of peripheral blood and incidence of CRC. In one study, the DNA methylation changes were found in genes associated with control of metastasis (AOX-114 and RARB215), angiogenesis (RERG16 and ADAMTS917), and autoimmunity (IRF4 and FOXE-1).^108^ Hypermutation of FOXE-1 had been earlier reported to be a predictive biomarker of CRC in biopsies obtained by colonoscopy.^109^ In another study, hypermutated promoter regions of a panel of 30 CRC-associated genes was assayed in circulating DNA and proposed to be a biomarker of CRC.^110^ To what extent these blood markers are derived from circulating tumor cells, versus PBMC, is yet to be determined. However, changes in the gut microbiota that are associated with distinct pathologies other than cancer, such as obesity, also translate into distinctly altered global peripheral blood mononuclear cells (PBMC) DNA methylation patterns.^111^ Thus, it is highly likely that microbiota regulate host immunity by altering methylation patterns of circulating immune cells.

It is very tempting to consider how microbiota-induced changes in the methylation patterns of immune cells can contribute to the nature of antitumor or pro-tumor immunity in CRC. GM have been proposed to contribute to immunological failures, which lead to disease predisposition and tumor growth^112^, as well as to effective immune surveillance and response to therapy.^113^ Numerous studies have established the role of GM in the maturation of the immune compartment of developing mammals and in determining the nature of immune responses in adults. Indeed, immune response is typically associated with alterations in DNA methylation. For example, the response of cancer patients to neoadjuvant chemotherapy^114^ or peptide vaccination^115^ can be monitored by changes in the patterns of DNA methylation in their PBMC. Altered methylation patterns of circulating CD8^+^ T-cells have been associated with expression of PD1 and T-cell exhaustion, as reviewed by Emran and colleagues.^39^ DNA methylation patterns define a population of anergic CD4^+^ effector T-cells that can differentiate into regulatory T-cells (Tregs).^116^ A study of the PBMC of pregnant women found an association between the differential methylation of promoter regions of gene associated with cardiovascular disease and Firmicutes and Bacteroidetes but not Proteobacteria.^117^

### Perspectives

We hypothesize that microbes and colonocytes develop adaptive processes to deal with their environment. By characterizing microbiota dysbiosis, we can establish a combined risk factor per individual that includes environmental and host-adaptive processes. The genetic mutations and epigenetic changes including DNA methylation that control gene functions are the main markers of such adaptive processes. Thus, we propose a holistic evaluation of CRC risk and outcomes that is based on individual environment-related risk factors, their impact on GM, and the methylation pattern of host tissues, including blood. Guan et al.^118^ characterized these risks for breast cancer incidence by evaluating and comparing the predictive performance of whole‐blood DNA methylation and genetic and environment risks in a prospective cohort with up to 14 years of follow‐up. They showed that all three types of risk scores were predictive of breast cancer occurrence. In their study, a genetic risk score that was based on multiple common variants (269 SNPs) predicted cancer incidence with much higher accuracy than either methylation risk that was based on previously identified CpGs or an environmental risk score that was derived from previous studies. They also showed that the combination of three risks enhanced risk prediction, with an “area under the cure” (AUC) statistic value of approximately 0.64. Similar predictive accuracy of either individual or combined risk scores was observed in specific subgroups that were defined by time to diagnosis. We believe that microbiome analysis should improve environmental risk markers and can help to identify CpG methylation targets that relate to cancer risk. Thus, physicians may validate a combination of microbial and molecular (genetic and epigenetic) markers to diagnose tumors and to predict related prognosis.

Whether microbiota exerts a direct or indirect effect on the epigenome remains unclear. Nevertheless, it is likely that much of the impact of microbiota on the tumor is through modulation of the immune response. In addition to bacterial products, such as LPS, the metabolism of nutriments and bile acids by bacteria impact the host’s innate and adaptive immunity. These include short-chain fatty acids (SCFAs), hydrogen sulfide (H_2_S), and secondary bile acids, all of which alter the genome or epigenome of immune cells.^78^ The SCFAs (acetate, propionate, and butyrate) enhance the induction of Tregs and certain bile acids, while bile acid metabolites alter inflammation by inducing the expression of the canonical T helper 17 transcription factor and retinoic acid-related orphan receptor gamma (RORγt) in the Tregs,^119^ and serve as ligands to RORγt.^120^ Future research will establish the role of bacterial metabolites in determining the fate of immune cells through DNA methylation.
